# Do Meio- and Macrobenthic Nematodes Differ in Community Composition and Body Weight Trends with Depth?

**DOI:** 10.1371/journal.pone.0014491

**Published:** 2011-01-06

**Authors:** Jyotsna Sharma, Jeffrey Baguley, Bodil A. Bluhm, Gilbert Rowe

**Affiliations:** 1 Department of Biology, University of Texas at San Antonio, San Antonio, Texas, United States of America; 2 Department of Biology, University of Nevada, Reno, Nevada, United States of America; 3 Institute of Marine Science, School of Fisheries and Ocean Sciences, University of Alaska Fairbanks, Fairbanks, Alaska, United States of America; 4 Department of Marine Biology, Texas A&M University at Galveston, Galveston, Texas, United States of America; University of Plymouth, United Kingdom

## Abstract

Nematodes occur regularly in macrobenthic samples but are rarely identified from them and are thus considered exclusively a part of the meiobenthos. Our study compares the generic composition of nematode communities and their individual body weight trends with water depth in macrobenthic (>250/300 µm) samples from the deep Arctic (Canada Basin), Gulf of Mexico (GOM) and the Bermuda slope with meiobenthic samples (<45 µm) from GOM. The dry weight per individual (µg) of all macrobenthic nematodes combined showed an increasing trend with increasing water depth, while the dry weight per individual of the meiobenthic GOM nematodes showed a trend to decrease with increasing depth. Multivariate analyses showed that the macrobenthic nematode community in the GOM was more similar to the macrobenthic nematodes of the Canada Basin than to the GOM meiobenthic nematodes. In particular, the genera *Enoploides, Crenopharynx, Micoletzkyia, Phanodermella* were dominant in the macrobenthos and accounted for most of the difference. Relative abundance of non-selective deposit feeders (1B) significantly decreased with depth in macrobenthos but remained dominant in the meiobenthic community. The occurrence of a distinct assemblage of bigger nematodes of high dry weight per individual in the macrobenthos suggests the need to include nematodes in macrobenthic studies.

## Introduction

Free-living nematodes are an abundant and diverse phylum that is usually considered in studies of meiobenthos. While several macrobenthic studies note their presence, they are identified only to phylum level and not considered in macrobenthic assemblages [1]. A comparison of body weight in individuals of different taxa showed that nematodes had the highest percent carbon dry weight suggesting an important role in the carbon cycle [2]. The main reason for the omission of large nematodes in macrobenthos studies is that the distinction between macro- and meiobenthos was originally based purely on sieve size [3]. Meiobenthos are defined as fauna retained on 42/63 µm sieves [4] and macrobenthos are defined as, depending on habitat, fauna retained on 0.25–1 mm sieve apertures [5]. A more flexible definition of these groups is now widespread and is based on taxonomic composition [6]. Macrobenthos *sensu stricto* excludes nematodes, harpacticoids and ostracods [4]. Large nematodes are, therefore, hardly ever taxonomically identified in any study from a given region.

The following trends on biomass and nematode size variation with water depth have emerged from numerous studies. Both total macrobenthic and meiobenthic biomass generally decreases with water depth though the rate of decline is greater for larger organisms [7]. This decline is partially explained by the decrease in average metazoan size with water depth [8]. The size of individual nematodes has also been noted to decrease with depth in the deep sea [9], [10] and has been attributed to the typically decreasing availability of food and decreasing sediment grain size. Studies on the functional diversity of nematodes as determined by buccal morphology show that with increasing water depth deposit feeders predominate while predators were less dominant [10]. However, macrobenthic nematodes have not been included in these studies and our current study emerged from an interest in evaluating the role of these larger nematodes in benthic communities and food webs.

Here we test the null hypothesis that meio- and macrobenthic nematode communities do not differ in structure and function. Specifically, the structure of the nematode community was examined by (1) measuring average weights of individual nematodes in macrobenthic samples from the Gulf of Mexico, high Arctic Canada Basin and Bermuda transect and in meiobenthic samples from the Gulf of Mexico in relation to water depth, and (2) comparing the community composition of macrobenthic (GOM, Canada Basin, Bermuda transect) and meiobenthic (GOM) nematodes using a multivariate approach. The functional role of meio- and macrobenthic nematodes was determined by comparing the feeding group distribution based on their buccal morphology. Taxonomic and abundance data for the Canada Basin can be found in Sharma and Bluhm [11] and the GOM and Bermuda transect data are not yet published.

## Methods

**Figure 1:**
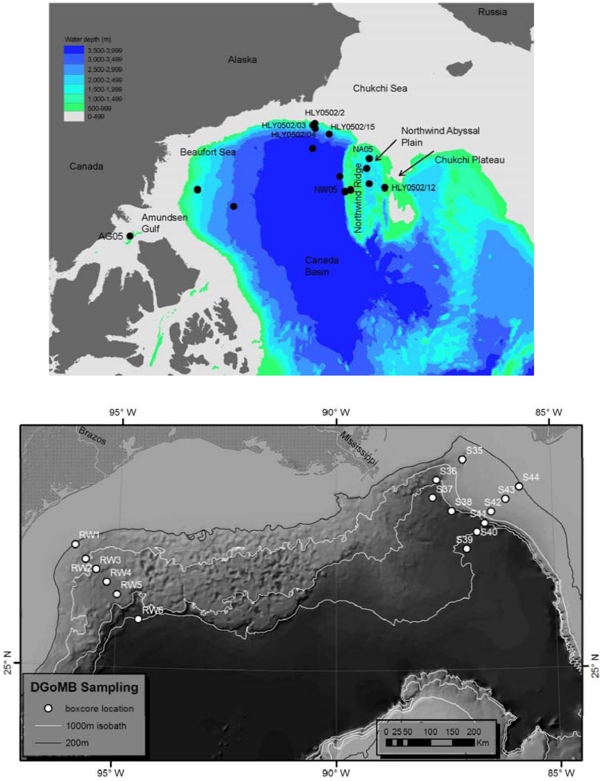
Sampling locations. A. Canada Basin. B. Gulf of Mexico (from Wei et al. [34] revised by Chih-Lin Wei).

Meio- and macrobenthic samples were collected from the Gulf of Mexico with a GOMEX boxcorer (0.2 m^2^) during May and June 2000 (Figure 1) [12]. 28 samples were collected from 14 stations in water depths of 212–3000 m. A set of five subsamples were set within the box thus reducing the total area sampled for macrobenthos to 0.17 m^2^. Sediments were removed, along with overlying water, down to a depth of 15 cm within the box and sieved on a 300 µm sieve. The meiobenthic samples were two of the seven subsamples with the box, each with a 5.5 cm inner diameter and a circular surface area of 22.9 cm^2^. The meiofauna were extracted by sieving through a 45 µm mesh sieve and centrifugation with Ludox [9].

Macrobenthos from the Arctic deep-sea Canada Basin were collected from a total of 22 quantitative box corer casts at a total of 8 stations at depths of 640–3961 m (Figure 1). The samples were collected with three replicates at each station in 2005 with 0.06 m^2^ surface area for replicates 1 and 2 and 0.03 m^2^ for replicate three [13], and mostly without replication in 2002 (0.04 m^2^ surface area) [1]. The top 10 cm of sediment and the overlying water was sieved through a 250 µm sieve. Details about the study area and additional benthic collections can be found in Bluhm et al. [1] and MacDonald et al. [13].

The Bermuda Slope samples were collected with an epibenthic sled at 1535–2200 m depth with details on sampling locations and methods in studies of Sanders and Hessler [14]. The samples examined are deposited at the Museum of Comparative Zoology, Harvard University, Cambridge, MA. The GOM and Canada Basin samples will be archived at the National Museum of Natural History, Washington, D.C.

Samples from all areas were preserved in 4% buffered formalin and later transferred to 70% ethanol. Nematodes were sorted by hand under WILD M3 and Leica M12 stereo-microscopes and processed to glycerin [15] for identification. All nematodes were identified to genus rather than species as many deep-sea species are not described. The total length and width of ethanol-preserved nematodes were measured on a Zeiss Axioscop to the nearest 20 µm. Five representative individuals (juveniles and adults) of each genus were measured to obtain average measurements. Biomass was calculated by the formula: Wet Weight =  Length × Width^2^/1600000 [16]. Dry Weight was calculated as 25% of wet weight [17].

Multivariate community analysis was carried out using PRIMER™ version 6 [18]. Bray-Curtis similarity was calculated on the abundance matrix after presence-absence transformation. Similarities between station groups were tested using analysis of similarities (ANOSIM) in which global R = 1 indicates complete separation of groups and global R = 0 indicates no separation [19]. A similarity profile test (SIMPROF) was performed on group average cluster analysis to test the null hypothesis that the macro- and meiobenthic samples do not differ from each other.

The functional diversity of the nematode community was analyzed by classification into one of four feeding groups: Selective deposit feeders (1A), non-selective deposit feeders (1B), epigrowth feeders (2A), predators and omnivores (2B). Though there have been further divisions of these feeding categories [20] the original scheme introduced by Wieser [21] is used here.

## Results

In all study areas combined, 177 nematode genera were found representing 38 families. Detailed lists and abundances in for the Canada Basin are included in Sharma and Bluhm [11]. 128 nematode genera occurred in the GOM meiobenthos while 60 genera occurred in the GOM macrobenthos (Sharma and Baguley, unpublished). There were 75 genera from 25 families in the Canada Basin samples (ibid.). The Bermuda slope transect had 15 nematode genera from 8 families (Sharma and Baguley, unpublished).

**Figure 2:**
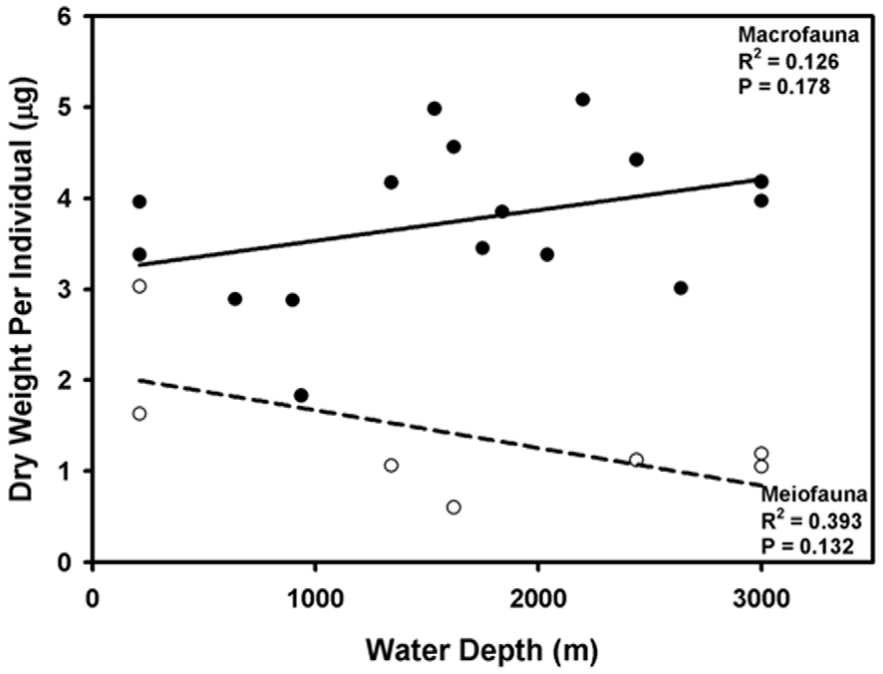
Station mean dry weight per individual of nematodes across genera from meio- and macrobenthos in the Gulf of Mexico, Canada Basin and the Bermuda transect.

Macrobenthic nematodes measured 400–8600 µm in body length and 14–130 µm in width, translating into a weight range of 0.04–7.34 µg per individual (2.11±2.70 µg; mean ± SD). Meiobenthic nematodes measured 300–3500 µm in body length and 25–110 µm in width translating into a weight range of 0.60–3.03 µg (1.38±0.78) per individual. The mean dry weights of individual macrobenthic nematodes showed a non-significant increase with water depth while the biomass of meiobenthic nematodes decreased non-significantly with increasing depth (Figure 2).

**Figure 3:**
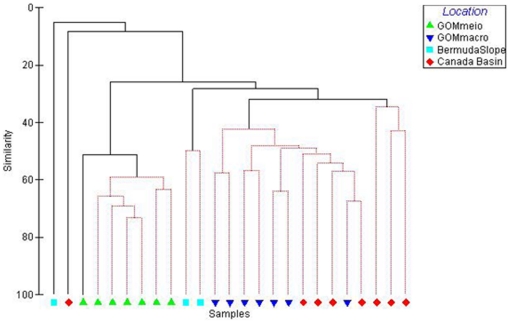
Hierachical cluster analysis with SIMPROF test on similarity of nematode genera from meio- and macrobenthos in the Canada Basin and the Bermuda transect based on presence/absence of genera.

Cluster analysis of nematode genera indicates that the macrofauna nematodes of the GOM were more similar to those of the Canada Basin and Bermuda slope than to the meiofaunal nematodes at the same GOM stations (Figures 3). High R values in the ANOSIM support the separation of GOM meio- and macrobenthic nematode communities while no significant separation was seen between the GOM and Canada Basin macrobenthic nematode communities (Table 1).

**Table 1:** Results of analysis of similarities (ANOSIM) of the Bray Curtis similarity matrices for meio- and macrofauna nematodes genera, family and feeding groups.

Pairwise comparison	R statistic	P-value
Genus Global R = 0.687, p = 0.001		
GOM meio vs macrofauna	0.995	0.003
GOM macrofauna vs CB macrofauna	0.261	NS
Family Global R = 0.513 p = .001		
GOM meio vs macrofauna	0.677	0.001
GOM macrofauna vs CB macrofauna	0.171	NS
Feeding Group Global R = 0.603, p = .001		
GOM meio vs macrofauna	0.908	0.001
GOM macrofauna vs CB macrofauna	0.351	NS

Footnote: GOM = Gulf of Mexico, CB = Canada Basin.

**Figure 4:**
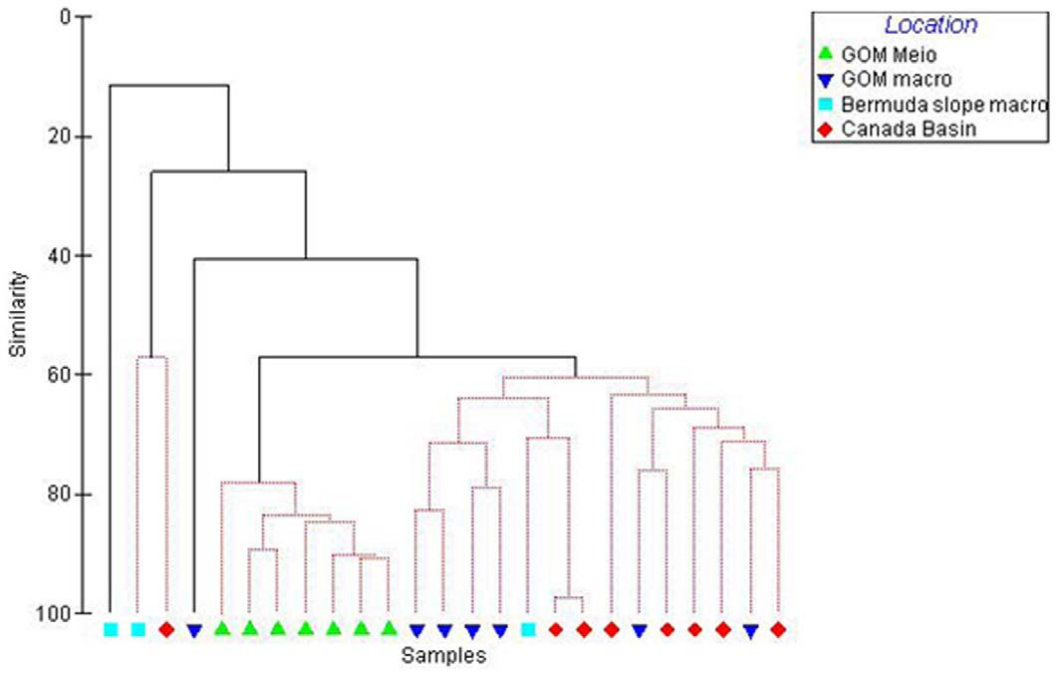
Cluster analysis with SIMPROF test on similarity of nematode families from meio- and macrobenthos in GOM and from macrobenthos in Canada Basin and Bermuda transect.

A SIMPER analysis indicated 73% dissimilarity between the meio- and macrofauna nematode genera in the GOM with genera such as *Enoploides, Crenopharynx, Micoletzkyia* and *Phanodermella* present in macrobenthic samples and accounting for part of the difference (combined 7%). These larger nematodes that were predominant in the GOM, Canada Basin and Bermuda slope occurred rarely in the meiobenthic samples. The highest within-area similarity was found in the GOM meiofauna nematodes (60%) with genera such as *Halalaimus, Desmoscolex, Microlaimus* and *Ammotheristus* contributing most to this similarity (combined 17%). The pattern did not change greatly when the analysis was run at the family level (Figure 4).

**Figure 5:**
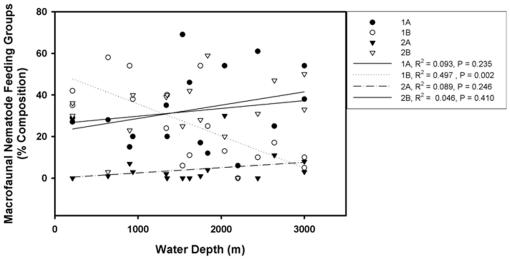
Relative composition of nematode feeding groups in pooled macrobenthos samples of GOM, Canada Basin and Bermuda transect.

**Figure 6:**
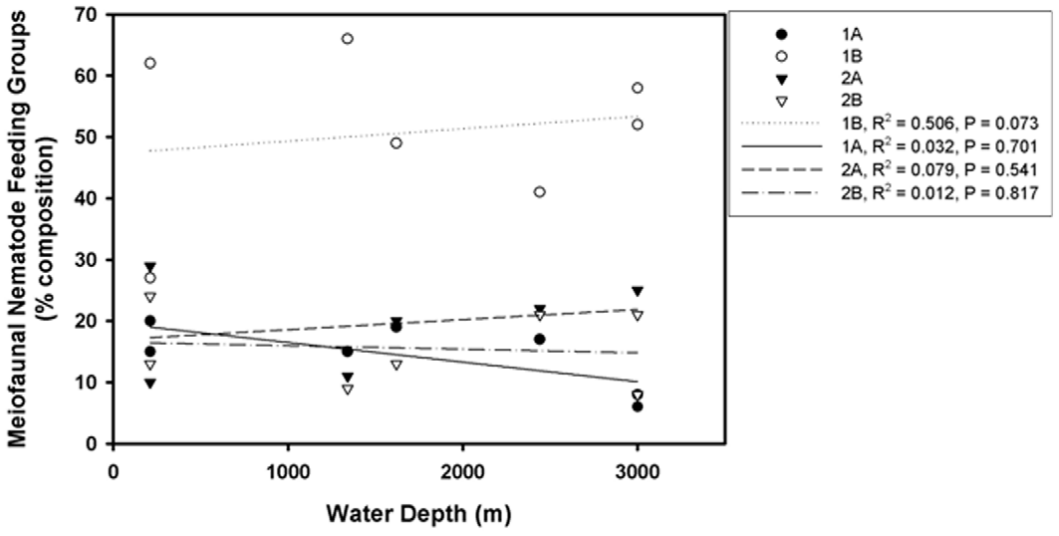
Relative composition of nematode feeding groups in meiobenthos samples of GOM.

The feeding group composition of the macrofauna nematodes from the GOM, Canada Basin and Bermuda transect combined shows a non-significant increase of predators and omnivores (2B) and selective deposit feeders (1A) with increasing water depth. Group 1B decreased significantly with depth in macrofauna (p = 0.002, Figure 5) but remained dominant in the meiofauna community (Figure 6). In the meiofauna samples, feeding group composition did not change significantly with depth nor were there any obvious trends, though as noted above, the non-selective deposit feeders remained dominant at all depths. The dominant feeding group in both the meiofauna and macrofauna communities was 1A. Group 2A was the least represented feeding group in the macrofauna sample. The slight increase of epigrowth feeders with increasing water depth in the meiofauna samples is due to increased presence of Desmodoridae genera. Group 1B was abundant at all stations and represented by larger genera of the Comesomatidae (*Sabatieria*) and Phanodermatidae (*Micoletzkyia,* and *Phanodermella)*. Group 2B was only present at some deeper stations of the Canada Basin.

**Figure 7:**
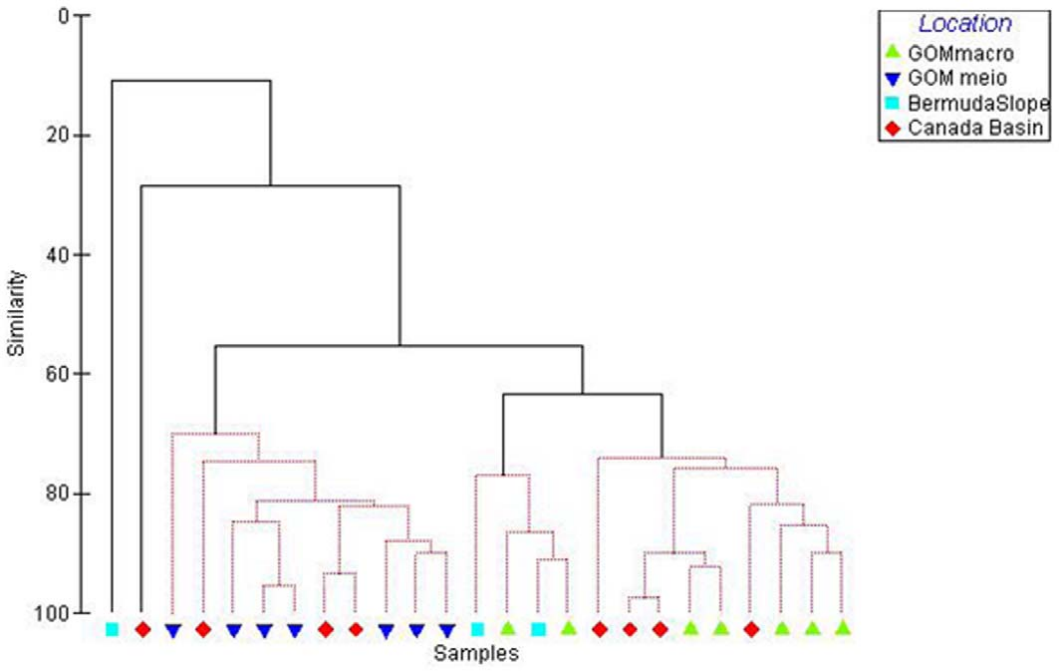
Cluster analysis with SIMPROF test on similarity of nematode feeding groups from meio- and macrobenthos in the Gulf of Mexico (GOM) and from macrobenthos in Canada Basin and Bermuda transect.

The cluster analysis of nematode feeding groups from meio- and macrofauna defined three significant clusters with meiofauna all grouping in one cluster but including three Canada Basin stations (Figure 7). High R values in the ANOSIM again supported the separation of GOM meio- and macrobenthic nematode communities while no significant separation was seen between the GOM and Canada Basin macrobenthic nematode communities (Table 1). Again, the highest average within group similarity (81%) was in the feeding group composition of the GOM meiofauna (SIMPER analysis). The group 1A contributed 53% to this similarity. The highest dissimilarity between groups was between the GOM meio- and macrofauna.

**Figure 8:**
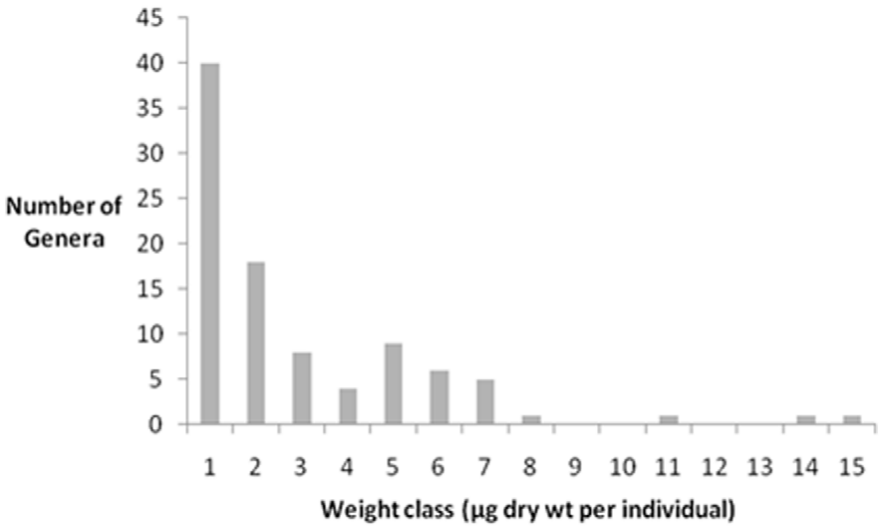
Numbers of nematode genera in each weight class.

A frequency distribution of individual body weight suggests a bimodal distrbution with one mode including the meiobenthic nematodes and the second comprised of macrobenthic nematodes (Figure 8). There are fewer genera among the larger weight classes that comprises nematodes of 4 µg to 15 µg dry wt per individual than among the 1–3 µg dry wt per individual weight classes.

## Discussion

### 1. Size and biomass

Meiobenthic nematodes are defined as such by size and are logically smaller than macrobenthic nematodes. The plot of nematode sizes shows a predominance of smaller nematodes that are part of the meiofauna (Figure 8). The meiobenthic and macrobenthic faunas of shallow subtidal regions are characterized by a defined bimodal distribution of body sizes [22]. In the deep sea, however, this distinction between the meio- and macrobenthos body sizes is not well defined [23]. Nevertheless, the depth trend in our data, although non-significant, combined with the depth frequency distribution, may suggest a size gap between meio- and macrobenthic nematodes in the deep sea areas examined.

Larger-bodied nematode genera such as *Crenopharynx, Micoletzkyia* and *Phanodermella* and families such as Leptosomatidae, Thoracostomopsidae and Phanodermatidae dominated our macrobenthos samples at greater depths and are seldom recorded in meiobenthic studies. The relationship of our GOM meiofaunal nematodes to water depth, although not significant, probably due to small sample size, supports the widespread trend of decreasing meiofaunal nematode body weight with increasing water depth [8], [7], [16]. The occurrence of short, stout nematodes, such as Desmoscolecids, at shelf break sites and long, slender nematodes in deeper waters has been noted in several meiobenthic studies [10]. This miniaturization of nematodes in the deep sea has been found across latitudes including North-Eastern Atlantic, Belgian continental shelf, Mediterranean and Indian ocean. It is attributed to decreasing food supply at greater depths [24] and follows the trend observed for macrobenthos [25].

In contrast, our observed possible *increase* in macrofaunal nematode size with depth agrees with the only reported exception to the miniaturization for nematodes: in the Western Pacific the median size of nematodes increased with increasing depth, though there was a significant decrease in the median size of all meiobenthos combined [26]. This author attributed the increase to an adult-rich and juvenile-rare population structure at greater depths. This was, however, not the case in our study (data not shown) and in others [16] that found a high abundance of juvenile nematodes of all taxa.

The upper limit to the size of meiofauna has been suggested to be 0.5–1.0 mm [27] as the organisms would shift from an interstitial to burrowing lifestyle. Although not tested in this study, environmental factors such as sediment properties, trophic interactions, and sampling factors such as fixation method and sieve size influence body size [11] and the environmental factors are related to depth [24]. The finer sediments found at deeper water depths may limit interstitial organism size. Thus long thin nematodes such as *Halalaimus* that can move through finer sediments are well represented in deeper waters in both meiobenthic and macrobenthic fauna. These thin nematodes are also well represented in meiobenthic studies as they pass through the 1.0 mm sieve [16]. Warwick [22] suggests that meiofaunal traits such as feeding and resource partitioning are optimized at 45 µg dry weight body size. The larger size may facilitate burrowing and overcome movement barriers in the finer sediments in deeper waters [27]. The smaller flocculents generally reported at greater water depths may also allow for less restricted movement at greater water depths. The only published record of macrobenthic nematodes apart from ours is from a polluted river where they were associated with fine sediments [28] but were absent in coarse sediments.

The choice of sorting methods to extract nematodes may also affect their observed size in benthic studies that do not use an upper size limit to extract meiofauna. A study in the Gulf of Mexico found that manual sorting produced more taxonomic groups and higher abundance of nematodes than the traditional extraction method by Ludox centrifugation which removed the larger nematodes of the macrobenthos [29]. The biomass of hand-sorted nematodes was also significantly higher in samples from the abyssal sites. Further analysis of the size ranges of the nematodes could determine if this reflects higher biomass of abyssal nematodes that may constitute macrobenthos.

### 2. Community structure

The meiobenthic nematode community in the deep GOM was found to be significantly different than macrobenthic nematodes from the same and two other deep sea regions, while the macrobenthic nematodes from these three regions did not significantly differ from each other (Table 1). These families of macrobenthic nematodes, namely, Phanodermatidae and Leptosomatidae are seldom recorded in meiobenthic nematode studies. The generic composition of the meiobenthic samples is similar to that of other studies with a dominance of Comesomatidae and Xyalidae [10]. The large dissimilarity between nematode genera contributing to meiobenthic and macrobenthic communties observed here is a significant finding and supports the theory that meiofauna and macrofauna are functionally separate communities [27]. Our study is the first record of several genera from the GOM and western Atlantic, namely, *Thoracostomopsis, Micoletzkyia, Phanoderma, Phanodermella* and *Synonchus* as they have not been considered in previous meiobenthic studies in these regions [30]. Similarly, at least seven genera found in the Canada Basin samples were new records for the Arctic deep sea [11].

### 3. Functional groups

Among the macrobenthic nematodes, the observed trend of increasing proportion of predators and omnivores, such as Oncholaimidae and Enchelidiidae at increasing depth may be related to reduced competition by other macrobenthic predators. Omnivory is also a useful trait in the deep sea where food is scarce. These families are almost absent from deeper waters in studies of meiobenthic nematodes [31], [32]. The selective deposit feeding families Phanodermatidae and Leptosomatidae apparently predominate at greater depths and displace the non-selective deposit feeders, Monhysteridae and Comesomatidae that are prevalent at the shallower water depths. While the classification of marine nematodes into feeding groups is based solely on stoma structure, observations in the lab have shown that nematodes are flexible in feeding preferences [33]. However, given the significant reduction of selective deposit feeders in the macrobenthic community but dominance of this feeding group in the meiofauna community, we interpret these data as further support of the conclusion that the meiobenthic and macrobenthic nematodes constitute structurally and functionally different communities.

### 4. Conclusions

Data presented here support the idea that meio- and macrofauna represent two unique communities, rather than one continuous community within a taxonomic group (e.g. Nematoda) as evidenced by: 1) different community structure (meio- vs. macrobenthic nematodes), 2) different body sizes, regardless of depth trends, and 3) the different functional response with depth as evidenced by the loss of the dominance of non-selective deposit feeders (1B) among macrobenthos.
